# Whole Brain Imaging with Serial Two-Photon Tomography

**DOI:** 10.3389/fnana.2016.00031

**Published:** 2016-03-22

**Authors:** Stephen P. Amato, Feng Pan, Joel Schwartz, Timothy M. Ragan

**Affiliations:** ^1^TissueVision, Inc.Cambridge, MA, USA; ^2^Pfizer, Inc.Cambridge, MA, USA

**Keywords:** serial two-photon tomography, serial section tomography, brain mapping, neural circuits, Alzheimer’s disease

## Abstract

Imaging entire mouse brains at submicron resolution has historically been a challenging undertaking and largely confined to the province of dedicated atlasing initiatives. This has limited systematic investigations into important areas of neuroscience, such as neural circuits, brain mapping and neurodegeneration. In this article, we describe in detail Serial Two-Photon (STP) tomography, a robust, reliable method for imaging entire brains with histological detail. We provide examples of how the basic methodology can be extended to other imaging modalities, such as Optical Coherence Tomography (OCT), in order to provide unique contrast mechanisms. Furthermore, we provide a survey of the research that STP tomography has enabled in the field of neuroscience, provide examples of how this technology enables quantitative whole brain studies, and discuss the current limitations of STP tomography-based approaches.

## Introduction

Over the last two decades there have been dramatic improvements in the fields of microscopy, tissue labeling, and computational image analysis. Each of these fields address an important aspect of quantitative biological imaging: optical microscopy methods visualize tissues and map the spatial-temporal relationship between components too small to be seen by the naked eye; tissue labeling techniques establish contrast and biochemical specificity of tissue components; computer-aided image analyses quantitatively explore relationships between the various components and makes it possible to handle the vast amounts of data that is generated (Klunk et al., 2002; Dean and Palmer, 2014; Piccinini and Shagrir, 2014; Jordan and Mitchell, 2015; Feng et al., 2015). The combination of advances in these three disciplines have a sum greater than the individual parts and particularly benefit the field of neuroscience, where researchers studying the central nervous system (CNS) are attempting to decipher a complex and enormous 3D network of interconnected neural circuits and cell types.

The three dimensional (3D) structure of the CNS (Bourdenx et al., 2014) is critical for proper network formation and function and therefore has been intensely studied. Traditionally, these studies have relied upon serial section analysis to investigate 3D cytoarchitecture of the CNS. In this technique, a tissue is manually sectioned into thin slices on a cryostat before the tissue section is mounted onto a microscope slide, stained and imaged using a microscope. For studies attempting to construct a 3D representation of the original tissue, the individually imaged sections must be aligned and digitally reassembled. However, as this method requires that tissue samples be sectioned prior to imaging, irreducible distortions are introduced into the tissue sections that preclude accurate full 3D reconstructions of the tissue. Furthermore, as this process is performed manually, the large datasets, required to generate robust and conclusive data, pose challenges both in terms of the labor and cost involved.

To circumvent some of the limitations imposed by serial section analysis, block-face approaches have been developed. In block-face imaging, the surface of the tissue block is removed using a microtome and the newly exposed tissue surface is imaged (Ewald et al., 2002; Denk and Horstmann, 2004). This process is then repeated throughout the tissue volume at fixed increments to achieve whole organ imaging. While less labor intensive than serial section analysis there are several limitations. First, sectioning the surface of the block introduces unavoidable distortions onto the surface. Since the newly exposed tissue has yet to be imaged, these distortions can corrupt the data. Second, since the axial resolution of the dataset is determined by the thickness of the slice, high resolution datasets will require numerous physical sections to be made which is time consuming and technically challenging. Finally, scattered or fluorescent light from layers below the block surface will distort the surface image, thereby reducing effective axial resolution and contrast (Krishnamurthi et al., 2010).

To address these limitations, we have developed a robust alternative that allows for a high degree of automation while maintaining sub-micron resolution and compatibility with current histological preparations, fluorescent proteins and dyes. While our approach, referred to as Serial Two-Photon Tomography (STP tomography; Ragan et al., 2007, 2012), is based on two photon microscopy (TPM), the methodology is applicable to a variety of 3D microscopy techniques. Here, we describe the key features of STP tomography and provide examples of research currently utilizing the method.

## Serial Section Tomography

STP tomography is based upon the basic methodology of alternating 3D imaging and physical sectioning and while our approach utilizes TPM, this technique is compatible with a variety of 3D imaging modalities. Examples include optical coherence microscopy, second harmonic generation (SHG), third harmonic generation (THG), coherent anti-stokes Raman scattering, and stimulated Raman scattering (SRS). Therefore, to distinguish between STP Tomography and the more general case, we will use the term Serial Section Tomography (SST) for the remainder of this technical report.

**Figure 1:**
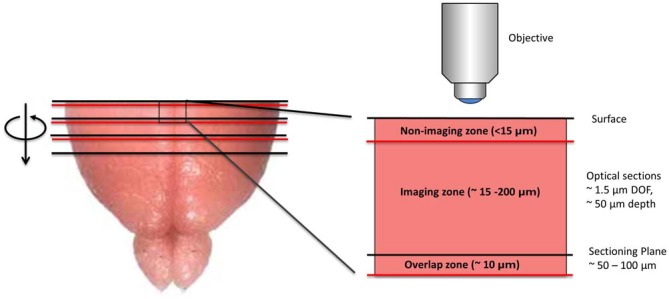
**Diagram depicting the imaging methodology employed by serial section tomography (SST).** The uppermost portion of the brain is sectioned using a vibratome and removed from the tissue block. The newly exposed tissue surface is imaged anywhere from 15 to 200 μm below the surface, thereby avoiding sectioning artifacts. Two-photon applications allow single layers or *z*-stacks to be acquired within the imaging zone of the tissue. While the imaging depth is usually restricted to 200 μm below the tissue surface to maintain sufficient contrast and resolution, deeper sections can be imaged in cleared or transparent tissues. Section thickness typically ranges from 50 to 100 μm, as this allows captured tissue slices to be used in additional investigations, including immunohistochemical analysis. If full 3D tissue reconstruction is desired, volumetric stacks extending past the sectioning depth can be acquired, stitching successive volumes together axially to form full 3D images.

**Figure 2:**
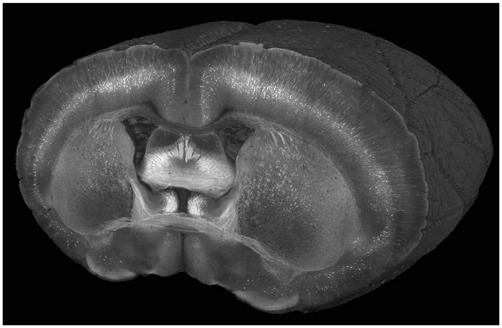
**A cutaway view of a Thy1-YFP-3204 mouse brain imaged at a wavelength of 900 nm with a coronal spacing of 100 μm and an *xy* resolution of 1.2 μm**.

SST has two key technical features that distinguish it from serial section analysis and block-face imaging. First, unlike serial section analysis, the tissue section of interest is always imaged before it has been removed from the tissue block. Second, as opposed to traditional block-face imaging, the imaged portion of the tissue is tens to hundreds of microns below the surface of the block and not at the block surface (Figure 1). Taken together, these two features offer significant advantages. First, by imaging the section before it has been cut from the tissue block, SST can achieve near perfect registration between successive tissue sections. As shown later, this is of particular importance when constructing brain atlases. Further, since tissue images are acquired below the block surface, the tissue remains in a pristine state, free from the deformations resulting from mechanical sectioning. Moreover, by automating the alternating steps of 3D optical sectioning and mechanical sectioning, it is possible to rapidly image through large volumes of tissue with no user intervention (Figure 2). This procedure effectively transforms 3D histology from an error-prone, low-throughput undertaking to a high-throughput, high-fidelity discipline.

Below we provide a brief description of the main features of SST.

### Straightforward Specimen Preparation

Specimen preparation consists of a standard formaldehyde perfusion fixation that introduces the fixative transcardially, harnessing the endogenous vasculature to distribute the fixative evenly throughout the entire brain. In addition to preserving the brain, fixation acts to stiffen the tissue, thus ensuring even and consistent sectioning throughout the tissue volume. To prepare the tissue for imaging and sectioning, the brain is embedded in an agarose block to provide mechanical stability, which is then glued to a glass slide and placed in a water bath. The sample preparation procedure is thus straightforward, robust, and does not require specialized equipment or chemicals. Furthermore, the tissue samples can be imaged 24 h after perfusion fixation or stored indefinitely in phosphate buffered saline.

### Minimal Tissue Processing

Tissue processing is a critical step for histological preparations as the experiment is of little value if tissue processing distorts the very biology in question. Given that chromophores, particularly fluorescent proteins, are sensitive to harsh preparation protocols (Giepmans et al., 2006) and that tissue preparation methods may display dramatic effects on tissue morphology (Howat and Wilson, 2014) the proper choice of the fixative and downstream processing steps are crucial. Importantly, tissue imaging using SST does not necessitate optical clearing, and only requires that brains be fixed prior to imaging, thereby avoiding the use of additional chemicals and tissue processing procedures. While a variety of tissue fixatives are available, formaldehyde fixation remains the gold standard for maintaining tissue morphology and antigen preservation (Roberts et al., 1990).

### Automation and Robustness

Straightforward sample preparation, in combination with the automated, and robust nature of the acquisition, results in a minimal failure rate for SST data production. When problems do occur, they can usually be attributed to improper fixation or embedding of samples and subsequent poor sectioning of the relatively soft paraformaldehyde fixed tissue. This failure rate is significantly reduced when a perfusion fixation is performed vs. an immersion fixation. To further ensure a high success rate, a 24 h post fix in 4% paraformaldehyde will also improve results. In the end, the high reliability opens the door for large-scale studies involving tens to even thousands of unique samples, including behavioral conditioning paradigms or systematic investigations like the Allen Mouse Brain Connectivity Atlas, which involved over 1200 C57BL/6J mice individually injected with distinct viral tracers (Oh et al., 2014).

### Full 3D Reconstructions

As noted, full 3D reconstructions can be achieved by scanning overlapping volumes between successive sections. Volumes are constructed by adjusting the focus of the objective lens to image multiple layers within the tissue. After scanning the volume, the uppermost portion of the tissue is mechanically removed but at a depth that is less than the extent of the volume scan, thus leaving an overlap region (Figure 1). The next successive volume scan can then be overlaid with the previous to form a contiguous volume. Hence, the axial resolution is not determined by the thickness of the physical section, but by the axial resolution of the imaging modality. In the case of TPM, an axial resolution of approximately 0.8 microns can be obtained. A challenge associated with obtaining seamless 3D reconstructions with SST however is the fall-off in intensity with increasing imaging depth. Increasing the laser power as imaging depth increases helps offset this issue; however, very dense tissue can still pose a challenge. Two ways to address this is to combine very mild clearing techniques that increase the imaging depth to approximately a few hundred microns (Economo et al., 2016) or to simply reduce the thickness of the physical sections.

### High Resolution

**Figure 3:**
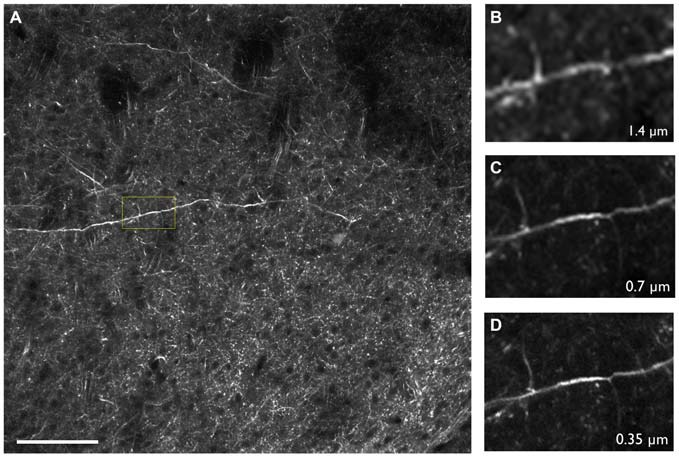
**(A)** Thy1-YFP-3204 transgenic mouse brain expressing YFP-positive neurons imaged using two-photon tomography.** (B–D)** Inset depicted in **(A)** enlarged and displayed with pixel sizes of 1.4, 0.7 and 0.35 μm respectively. Images were acquired using a 16× Nikon, 0.8 NA objective. Scale bar = 100 μm.

High resolution is critical for CNS studies as many key components of the brain are less than a micron in size, such as dendritic spines which require sub-micron resolution in order to be resolved (Adrian et al., 2014). SST is well suited for such applications as the methodology is capable of providing highly resolved images throughout the tissue. This is in part due to SST’s advantageous imaging geometry and the close proximity of a high numerical aperture objective lens to the brain surface. Furthermore, as the imaging depth commonly ranges from 25 to 100 microns below the tissue surface, there is little intervening tissue to produce light scattering effects. For these reasons, it is possible to achieve sub-micron resolution across the entire mouse brain with no degradation in resolution in the interior portions of the sample (Figure 3; Ragan et al., 2012; Economo et al., 2016). We note that even when imaging optically cleared tissues with TPM and long working distance objectives, significant degradation of image quality can be expected when imaging beyond approximately 2 mm into the tissue, due to the out-of-focus fluorescence that is generated. Thus, mechanically sectioning the tissue remains the best option to maintain sufficient resolution and contrast throughout the tissue volume (Richardson and Lichtman, 2015; Albanese and Chung, 2016; Economo et al., 2016).

### Specimen Size and Photobleaching

There is no fundamental limit to the size of the sample that can be imaged using SST. This feature is due to the fact that the imaging geometry employed by SST remains constant as tissue sections are sequentially removed. In comparison, light sheet microscopy relies upon lateral sample illumination and therefore must pass through the entirety of the tissue, ranging from millimeters to centimeters, in order to visualize interior compartments (Huisken and Stainier, 2009). Even with the best clearing techniques, the required tissue penetration depth broadens the light sheet, effectively reducing the optical sectioning capabilities of the approach and results in light scattering and aberration artifacts which reduce imaging resolution. To date, light sheet approaches have not been able to demonstrate consistent one micron resolution across samples more than a few millimeters in extent. In contrast, SST has displayed the ability to maintain near diffraction limited resolution across tissue samples centimeters wide. The main limitation to specimen size is dataset size and acquisition times, both of which we discuss in the limitations sections.

Non-linear imaging techniques such as multiphoton microscopy confer the advantage of limiting photobleaching outside the focal plane. The large tissue samples referenced above, extending centimeters in depth, can be imagined with no decrease in signal at deeper depths even after hours or days of extended imaging. Moreover, the individual image tiles used to assemble large high resolution images, can be constructed in a manner that discard photobleached overlapping regions in the final mosaic, effectively removing them from the dataset altogether.

### Specimen Preservation

While SST relies upon tissue sectioning, all of the sections are available for tissue collection and subsequent analysis. In contrast, other techniques such as 2p-fMOST require that the tissue be embedded into a hard resin and sectioned into thin strips which are difficult to recover, and susceptible to tears along the edges of the ribbon sections (Zheng et al., 2013). The tissue sections in SST are most often acquired at a thickness 25–50 microns, and are ideal for either free floating IHC labeling or a multitude of different analytical strategies, providing a significant advantage for many applications. For instance, researchers interested in performing genomic or proteomic investigations on SST processed tissue have the ability to analyze whole or partial tissue slices via RT-PCR, microarray analysis, imaging mass spectrometry or other biochemical means. In this way imaging investigations can provide information that is either difficult or impossible to obtain using current imaging-based approaches. Importantly, the resulting sections can be reimaged by employing additional imaging modalities such as confocal microscopy or wide field fluorescence. Information obtained from these modalities can easily be overlaid onto the original 3D data set to create rich, multimodal datasets (Van de Plas et al., 2015). Moreover, the flexibility afforded by having tissue sections indexed back to the 3D dataset allows investigators to later re-label and re-image tissue sections to address follow-on questions.

## SST Imaging Modalities

While fluorescence is an extremely valuable imaging modality, it does come with some practical limitations. First, given the wide emission spectrum of fluorescent dyes and proteins and the typical signal levels achieved by these labels, it is difficult to separate more than four components at once. This presents a problem when attempting to image multiple targets within a single sample. Second, it is often necessary to exogenously label the structure of interest with a fluorescent dye, which can be particularly difficult when dealing with thick tissue preparations. As such, label free imaging modalities based on endogenous signatures can provide extremely valuable complementary information that extend the reach of SST. By combining additional imaging modalities with SST’s standard imaging geometry and use of nonlinear excitation it is possible to produce feature rich datasets. Below we describe compatible imaging modalities.

### Two Photon Microscopy

**Figure 4:**
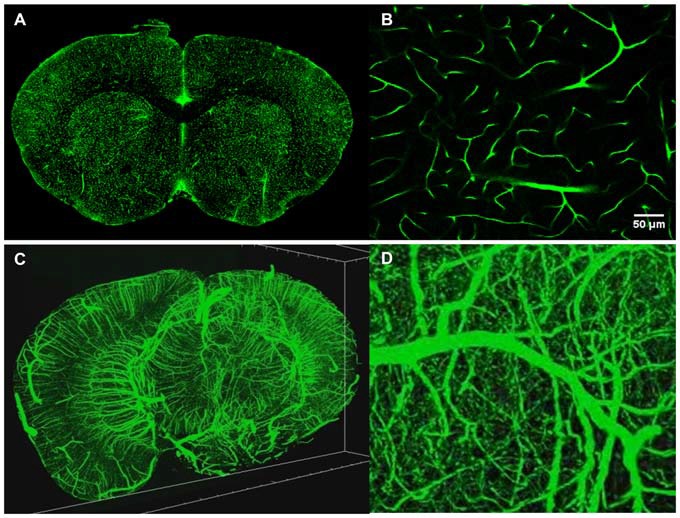
**Intravital vascular labeling of a whole mouse brain.** Following transcardial perfusion fixation, the mouse is perfused with FITC-conjugated gelatin, effectively filling the vasculature before cooling and solidifying. The FITC-signal provides a strong signal which can easily be isolated from the background using a global threshold approach. **(A)** Representative 2D coronal section of a C57/BL6 mouse labeled with FITC-conjugated gelatin. **(B)** Representative enlarged region of 2D coronal section similar to the one depicted in **(A)**. **(C)** 3D visualization of FITC-labeled mouse brain vasculature. 4 mm portion is shown. Resolution is 1 μm radial by 2 μm axial. **(D)** Representative enlarged region of 3D coronal section similar to the one depicted in **(C)**.

TPM is a high resolution 3D fluorescent imaging technique (Denk et al., 1990; So et al., 2000) and while the advantages of TPM are well documented for *in vivo* imaging, the technique also brings significant advantages to *ex vivo* imaging as well. First, due to the nonlinear nature of the excitation process, TPM provides inherent 3D sectioning, an excellent imaging depth of several hundred microns, minimal photobleaching outside the focal plane and works well with both opaque and cleared samples. The typical two photon excitation point spread function has a full width half maximum of 0.35 μm in the radial direction and 0.9 μm in the axial direction for tissue imaging at 920 nm with a 1.0 NA objective (Zipfel et al., 2003), a feature that becomes increasingly important when imaging axially extended samples such as whole brains. In addition, TPM has superior background rejection arising from the wide separation of excitation and emission wavelengths, and possesses the ability to excite multiple chromophores with a single excitation wavelength. Further, large field of views can be achieved with small depths of focus that are limited only by the objective optics. For instance, by perfusing FITC-conjugated gelatin throughout a C57BL/6 mouse brain after paraformaldehyde fixation (Tsai et al., 2009), we were able to utilize STP tomography to generate a three dimensional reconstruction of a portion of the cerebral vasculature (Figure 4). In contrast, the confocal parameter of the light sheet limits the field of view to often less than a few hundred microns, thus necessitating extensive tiling.

### Optical Coherence Microscopy

Optical Coherence Tomography (OCT) is a 3D optical imaging technique capable of imaging several hundreds of microns into biological tissues while producing high resolution images. OCT, originally introduced in 1991 (Huang et al., 1991), is an interferometric technique that combines low coherence interferometry and a broad spectral bandwidth light source to capture backscattered light of tissue samples. This methodology makes OCT sensitive to differences in the index of refraction within tissues and intrinsic contrast can be observed in cell bodies and myelinated fibers (Saxena and Jain, 2011).

**Figure 5:**
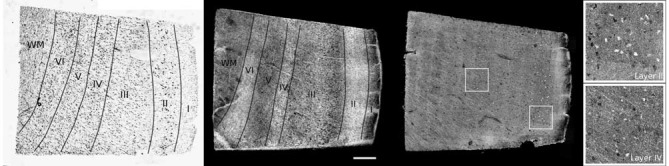
**Cortical layers of entorhinal cortex observed using Nissl staining (left) and maximum intensity projection of lower resolution optical coherence tomography (OCT; center).** Layers I to IV have been labeled, as well as the white matter (WM). The neurons are observed by OCT using a higher magnification (right). The insets show the neurons of layer II (top) and IV (bottom). Scale bar = 500 μm. Figure Credited from Magnain et al. (2015).

While OCT has traditionally been employed for *in vivo* imaging studies, multiple groups over the last several years have begun to demonstrate its utility for histopathology of the CNS (Goergen et al., 2012; Srinivasan et al., 2012; Magnain et al., 2014, 2015). Srinivasan et al. (2012) used high resolution OCT to image the cerebral cortex of the rat and successfully identified neuronal bodies and myelin sheaths surrounding axons. Magnain et al. (2014, 2015) imaged the human entorhinal cortex and compared the OCT images with traditional Nissl stained images of the same regions (Figure 5; Magnain et al., 2014, 2015). More recently, Wang et al. (2014) integrated a vibratome into a polarization sensitive OCT system and generated extended volumetric datasets of rat brains across several physical sections.

### Additional Modalities

Additional imaging modalities include, SHG, THG, coherent anti-stokes Raman scattering (Heneka et al., 2015), confocal reflected, and SRS. While it is beyond the scope of this article to describe each technique in detail, we point out that each of these is a label free method that is compatible with SST and does not require the introduction of exogenous dyes. For example, CARS microscopy can detect a wide array of brain structures, including myelin and fiber bundles (Evans et al., 2007), SRS has shown the ability to differentiate between healthy mouse brain tissue vs. tumor infiltrated tissue (Ji et al., 2013) and THG possesses the ability to visualize neurons, vasculature and white matter (WM) structures within the brain, showing particular sensitivity to lipid structures (Witte et al., 2011).

## Applications

The brain is unique in that it is composed of interconnected, yet functionally distinct, neuronal circuits that occupy spatially distinct sub-regions across the entire organ. For these reasons, systematic investigations attempting to elucidate the finer details of both intra- and inter-network communication have benefitted heavily from imaging techniques that provide spatial information containing all three dimensions (Guzowski et al., 2005). An SST approach provides the opportunity to examine multiple circuits in their entirety and simultaneously allows for the visualization of spatially defined cellular and morphological data. Here, we briefly touch upon a variety of applications where SST has been utilized to address fundamental questions in the field of neuroscience, as well as propose theoretical applications of the approach.

### Mapping Neuronal Activation

While SST can be characterized as a static methodology, relying upon fixed tissue for imaging, experimental techniques have been developed that allow SST to measure dynamic biological processes, including neuronal circuit activity. Specifically, through the utilization of fluorescently tagged immediate early gene (IEG) proteins, researchers are able to effectively monitor neuronal activation. IEG proteins, such as *c-fos* and *Arc*, undergo rapid and transient expression in response to neuronal activation and when expressed in transgenic animals as fluorescently conjugated proteins, can serve as reliable reporters for neuronal circuit activation in response to stimuli (Kovács, 2008; Eguchi and Yamaguchi, 2009). To-date, the majority of studies evaluating neuronal activity have relied exclusively upon fMRI and while this imaging modality provides both spatial and temporal resolution, it offers only gross macroscale resolution devoid of cellular or morphological information (Heeger and Ress, 2002; Logothetis, 2008). Conversely, whereas electrophysiology and *in vivo* imaging enable enhanced spatial resolution, these methods only allow for coverage over a small region of interest. The use of SST for *ex vivo* imaging, albeit lacking temporal resolution, overcomes the highlighted shortcomings of both fMRI and *in vivo* imaging by providing the ability to image entire brains at cellular resolution.

Underscoring this point, a study by Vousden et al. (2015) illustrated the ability of SST to perform *ex vivo* whole brain mapping of circuit activation in response to fear memory retrieval in transgenic mice expressing the IEG reporter, arc-venus. Specifically, SST was utilized to visualize arc-venus expression across the whole brain and the resulting high-resolution datasets were subsequently registered and annotated using the Allen Institute brain atlas. In combining whole-brain imaging with image registration and cell segmentation, researchers were able to determine the number of activated neurons per brain region in response to distinct behavioral stimuli. Importantly, this study identified specific neuronal networks in the amygdala, hippocampus and neocortex that are activated in response to fear memory retrieval, thereby demonstrating the utility of a SST approach.

The ability to map neuronal network activation using SST methodology, like the experimental paradigm highlighted by Vousden et al. (2015) is applicable to additional IEG transgenic animals. To this point, Kim et al. (2015) used a similar workflow to study behavioral-induced brain activation in a c-fos-GFP expressing transgenic mouse. Using the SST methodology in conjunction with computational image analysis, researchers performed a side-by-side comparison of female and male interaction-evoked whole-brain activation. Their results highlight distinct sex-dependent differences in the spatial organization of the circuits downstream of the medial olfactory bulb, leading to activation of distinct populations of neurons in the piriform and entorhinal cortex. The cellular data acquired on a whole-brain level, enabled researchers to quantitatively estimate neuron activation per brain region and ultimately correlate spatial activation patterns with specific features of social behavior. The results from this study again highlight the advantages that high-resolution whole-brain imaging affords systematic investigations attempting to dissect the relationship between specific behavioral stimuli and neuronal circuit activation.

### Mapping Neuronal Networks

A comprehensive understanding of the neuronal connections within the brain will provide us with invaluable insights as to how complex information is processed by the human brain and ultimately advance our understanding of both physiological and pathological brain function (Feng et al., 2015). However, our ability to map neuronal connections is dependent upon the ability to visualize and track neuronal cell bodies and their projections across multiple, anatomically distinct brain regions. To this point, SST is particularly well suited to satisfy the whole brain sub-cellular imaging required by such network mapping endeavors, providing whole-organ, high resolution imaging. Moreover, the high throughput capability of SST, combined with exceptional image registration, provides a significant advantage over alternative technologies.

In support of this notion, a recent study by Zapiec and Mombaerts (2015) has illustrated the ability of SST to successfully map axonal tracts in the mouse olfactory system. The mouse olfactory system consists of sub-populations of olfactory sensory neurons (OSNs) expressing the same odorant receptor type. These distinct groupings of OSNs project their axons to the olfactory bulb, where they converge onto a small number of glomeruli (Bozza et al., 2002). In order to precisely map the anatomical position of the glomeruli and their corresponding sub-populations of OSNs, researchers utilized genetically modified mice expressing multiple odorant receptor type specific axonal markers. Using a multiplexed approach, images acquired through SST were used to generate an anatomical 3D reconstruction of the mouse olfactory bulb, in which axonal tracts of fluorescently labeled OSNs were traced to their respective glomeruli. Using this approach, researchers quantitatively determined the degree of positional variability for a total of 352 glomeruli within the olfactory system of the mouse and determined that positional variability effectively correlates with odorant receptor subtype. This study elegantly illustrates the ability SST methodology to successfully map neuronal projections within the brain.

As previously mentioned, the highly automated, high resolution nature of SST methodology provides investigators with a unique avenue for investigating whole-brain connectomics. In fact, the Allen Institute, one of several worldwide groups focusing on brain organization, utilized SST to acquire high resolution whole-brain images and successfully map the mouse connectome on a mesoscopic scale (Oh et al., 2014). By utilizing a series of stereotaxic injections of GFP-expressing adeno-associated viruses (AAVs) into different brain regions, in combination with whole brain imaging, image registration, image processing and analysis, researchers were able to map the neuronal networks of the mouse brain at single-cell resolution (Kuan et al., 2015). The acquired high-resolution datasets with 0.35 μm *x*, *y* sampling and 100 μm *z* intervals totaling at 750 gigabytes per brain, were used to identify transfected neurons and trace their projections across whole mouse brains.

The automated and the high throughput capabilities of SST were key for reproducibly processing the 3000 animals used in the study, while the distortion-free images enabled the successful registration and annotation of the identified neuronal connection. The framework developed by the Allen Institute has been adopted by Japan’s Brain/MINDS project, where SST is currently being used to map neuronal networks within the marmoset brain on a macroscopic, mesoscopic and microscopic level (Okano et al., 2015).

### Gene Therapy

The promise afforded by advances in gene therapy has again placed AAVs at the forefront of drug discovery for neurological diseases (Foust et al., 2009; Bourdenx et al., 2014). For instance, in Parkinson’s disease, several gene therapy clinical trials have been tested using local injections of AAV2 into the striatum and subthalamic nucleus of PD patients, in hopes of maintaining or restoring dopaminergic neuron function (Eberling et al., 2008; Christine et al., 2009). Importantly, the discovery of BBB penetrant AAV serotypes presents a unique opportunity for CNS applications, where efficient and specific AAV distribution circumvents the need for highly invasive intracranial injections. However, while the repertoire of AAV serotypes demonstrating BBB penetrance has significantly expanded (Bourdenx et al., 2014), most clinical applications have been limited to the AAV2 (Mingozzi and High, 2011).

In order to take advantage of systemic administration of AAVs newly identified BBB penetrant AAV serotypes must be extensively profiled to protect against unwanted side effects resulting from nonspecific tissue distribution. Current biochemical practices, such as qPCR and ELISA, while capable of quantitatively analyzing genomic copy number or protein expression to determine AAV distribution, require manual dissection of individual brain regions to achieve modest spatial resolution. Additionally, data derived via biochemical analysis alone are devoid of any morphological and cellular information, thereby excluding critical information that may aid in the evaluation of potential therapeutics. Lastly, whereas traditional optical methods relying upon serial section analysis do in fact offer higher resolution and morphological data, the process of manually sectioning and imaging individual brain slices generally restricts these investigations to a fewer number of representative images across the brain.

For applications such as this, the automated and comprehensive imaging employed by SST could have a profound impact on the screening and characterization of brain-wide AAV distribution. For instance, utilizing an SST approach to perform systematic whole-brain imaging of mice injected with various serotypes of GFP-expressing AAVs will provide investigators with spatial and morphological rich information that can be used to accurately determine the distribution of AAV serotypes across the brain, allowing visualization of both individual cells and their projections. Based on a comprehensive whole brain distribution of uncharacterized and novel AAV serotypes, researchers will be able to effectively correlate specific AAV serotypes with their targeted brain regions, ensuring specific delivery of their gene of interest. This information will be of the utmost value as researchers evaluate the potential therapeutic and safety profiles of innovative AAV-based therapies.

### Alzheimer’s Disease and Amyloid Plaque Imaging

Accumulating evidence suggests that abnormal protein aggregation is central to Alzheimer’s disease (AD) pathology, affecting multiple neuronal cell types in functionally distinct networks throughout the brain (Dickerson and Sperling, 2008; Liu et al., 2010). Accordingly, these aggregates, composed of either beta amyloid (Aβ) or hyper-phosphorylated Tau proteins form plaques or neurofibrillary tangles, respectively, and commonly serve as a readout for determining the efficacy of novel therapeutics for AD. The importance of monitoring both the spatial and temporal dynamics of protein aggregation as they appear within specific brain regions is critical not only to understanding the molecular and physiological underpinnings of AD progression but also for calculating an individual patient’s response to potential therapies.

Researchers frequently rely upon biochemical methods, such as ELISA, for determining total brain Aβ concentration. While this approach provides quantitative data concerning the total concentration of Aβ across the brain, it is devoid of information pertaining to plaque size, shape or spatial patterning per brain region. Moreover, these methods fail to report on morphological changes that may occur as a result of treatment, including such parameters as the parenchymal vs. vascular plaque ratio as they lack the requisite resolution and contrast.

**Figure 6:**
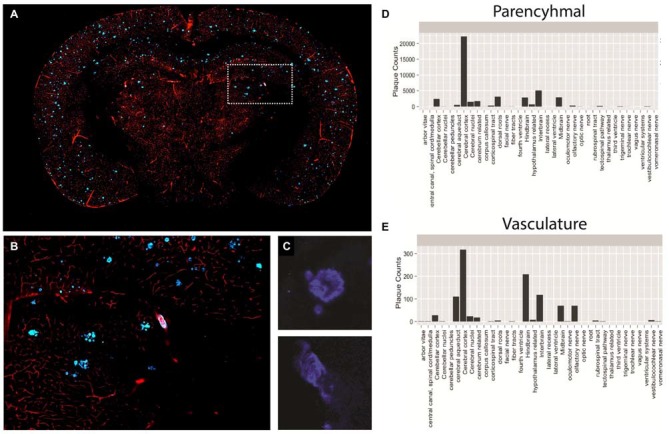
**Plaque and vasculature labeling in 3 month old amyloid precursor protein (APP)/PS1 mice. (A)** Representative coronal section of APP/PS1 mouse, exhibiting rhodamine positive vasculature and methoxy-X04 labeled plaques. **(B)** Inset depicted in **(A)**, enlarged to show a region of the methoxy-X04, rhodamine labeled brain slice in greater detail. **(C)** Methoxy-X04 signal for representative parenchymal and vascular plaques. **(D,E)** Classification and quantification of parenchymal and vascular plaques segmented by brain region. As described in the text, plaque type was determined by morphological shape and the spatial location of each plaque was determined by atlasing the whole brain image against the Ma 2008 atlas. The resulting information was used to classify plaque type and plot total plaque number against the individual brain regions.

For this reason, we performed a proof-of-principle experiment involving STP whole-brain imaging of a transgenic mouse model of AD harboring the amyloid precursor protein (APP) S1 mutation. The APP mouse possess a genetic mutation that results in enhanced Aβ plaque formation, exhibiting both parenchymal and vascular plaques, and is currently being used in AD drug discovery (Howlett, 2011). To evaluate both parenchymal and vascular plaques, we injected 3 month old APP mice with methoxy-X04, a systemically administered plaque-labeling compound, along with a fluorescently conjugated gelatin to effectively highlight the vasculature (Figures 6A–C). We then segmented all plaques across the brain with an adaptive threshold and classified each plaque as either parenchymal or vascular using an trained classifier (Shamir et al., 2008). The location of each plaque was then mapped against a standard atlas (Ma et al., 2008).

Using the described methodology we were able to successfully quantify both parenchymal and vascular plaques on a per region basis across the brain (Figures 6D,E). Our results illustrate a heavy plaque burden in the neo-cortex, consistent with previous studies investigating the APP mouse (Yan et al., 2009). However, by classifying plaque type, we effectively identified distinct trends of parenchymal and vascular plaque deposition across the brain. Specifically, while the neocortex of the APP mouse showed the greatest number of parenchymal plaques among the regions analyzed, the hindbrain contained the highest counts of vascular plaques. Findings of this nature may significantly impact treatments that target individual pathologies of complex neurological diseases and simultaneously reveal spatial differences in treatment efficacy.

## Limitations

While this review has discussed a number of applications that have benefitted from SST based approaches, the full potential of the described methodology remains limited by a number of factors. Firstly, many of the SST-based imaging approaches, including STP, rely upon a single focal point for sample scanning. While this limitation does not result in egregious imaging durations for atlas level imaging (1.2 μm *x*-*y* resolution and 100 μm sampling in the *z*-direction), processing an entire mouse brain in under 4 h, studies employing significantly higher sampling or much larger tissue specimens can quickly add hours to processing times. For instance, at current imaging speeds, an entire mouse brain at 1 μm × 1 μm × 2 μm sampling would take nearly a week of continuous imaging. However, there are several strategies that can be used to reduce this time. First, the imaging speed can be increased by integrating resonant scanners or scanning multiple foci in parallel (Bahlmann et al., 2007; Kim et al., 2007). These improvements can lead to an increase in the frame rate by at least a factor of 10. Additionally, selective imaging strategies can ameliorate long imaging times by focusing on specific regions of interest within a larger volume. While faster image times are feasible to achieve, a major hurdle faced by all whole brain imaging studies is processing the large amount of data generated. For instance, imaging a mouse brain with 15 μm spacing throughout the tissue volume using an SST-based approach can easily produce 1 terabyte worth of imaging data. With this in mind, researchers must establish a capable data transfer and storage infrastructure to accommodate large data volumes. Even with substantial IT infrastructure for data storage, the segmentation, registration and analysis of whole brain datasets will remain a significant challenge for the foreseeable future. Finally, while whole mount labeling strategies are currently being developed and improved (Renier et al., 2014; Kim et al., 2015), SST-based methods are currently most efficient when imaging samples that contain inherent fluorescence, achieved by either genetic modification or intracranial injection of fluorescently tagged components. While the SST approach does enable the option of performing IHC on post-SST processed tissue slices, additional work is required to produce a fully automated workflow from sample labeling to imaging. Once achieved, this approach will allow a multitude of biological readouts to be investigated across the brain as part of a fully automated and comprehensive image acquisition and analysis pipeline.

## Conclusion

In conclusion, STP tomography is a robust imaging technique that lends itself well to whole brain studies. The basic methodology can be expanded to include additional imaging and biochemical modalities leading to rich multi-component datasets. With a high success rate and minimal sample preparation, it is well suited for quantitative, large scale studies consisting of tens to hundreds of samples. In this review, we have highlighted a number of applications where the high optical and spatial resolution provided by SST has allowed researchers to assess global changes in numerous biological readouts. Furthermore, we provide a proof-of-concept experiment illustrating how an SST-based approach can be used to classify and quantitate Aβ plaques across multiple brain regions in a relevant mouse model of AD. Studies of this nature will help to provide a comprehensive understanding of clinical pathologies and pave the way for novel therapeutics.

## Ethical Statement

The animal work was done under TGA Sciences, Incorporated Instiutional Animal Care and Use Committee Vertebrate Animal Use. TGA Sciences, Inc. is located in Medford, MA, USA. The IACUC approval date was 5/21/2014 and the animal assurance number was A4497-01.

## Author Contributions

All authors listed, have made substantial, direct and intellectual contribution to the work, and approved it for publication.

## Conflict of Interest Statement

TMR and SPA are employees of TissueVision, Inc., which own the patents for serial section tomography and serial two-photon tomography and has commercialized the technology. The other authors declare that the research was conducted in the absence of any commercial or financial relationships that could be construed as a potential conflict of interest.
